# The Impact of Open Pollination on the Structural Evolutionary Dynamics, Meiotic Behavior, and Fertility of Resynthesized Allotetraploid *Brassica napus* L.

**DOI:** 10.1534/g3.116.036517

**Published:** 2016-12-21

**Authors:** Mathieu Rousseau-Gueutin, Jérôme Morice, Olivier Coriton, Virginie Huteau, Gwenn Trotoux, Sylvie Nègre, Cyril Falentin, Gwennaëlle Deniot, Marie Gilet, Frédérique Eber, Alexandre Pelé, Sonia Vautrin, Joëlle Fourment, Maryse Lodé, Hélène Bergès, Anne-Marie Chèvre

**Affiliations:** * Institut de Génétique, Environnement et Protection des Plantes (IGEPP), Institut National De La Recherche Agronomique (INRA), Université de Rennes 1, 35650 Le Rheu, France; † French Plant Genomic Resource Center (CNRGV), INRA, 31326 Castanet Tolosan Cedex, France

**Keywords:** *Brassica napus*, allopolyploidy, genome dynamic, meiotic behavior, fertility

## Abstract

Allopolyploidy, which results from the merger and duplication of two divergent genomes, has played a major role in the evolution and diversification of flowering plants. The genomic changes that occur in resynthesized or natural neopolyploids have been extensively studied, but little is known about the effects of the reproductive mode in the initial generations that may precede its successful establishment. To truly reflect the early generations of a nascent polyploid, two resynthesized allotetraploid *Brassica napus* populations were obtained for the first time by open pollination. In these populations, we detected a much lower level of aneuploidy (third generation) compared with those previously published populations obtained by controlled successive selfing. We specifically studied 33 resynthesized *B. napus* individuals from our two open pollinated populations, and showed that meiosis was affected in both populations. Their genomes were deeply shuffled after allopolyploidization: up to 8.5 and 3.5% of the C and A subgenomes were deleted in only two generations. The identified deletions occurred mainly at the distal part of the chromosome, and to a significantly greater extent on the C rather than the A subgenome. Using Fluorescent *In Situ* Hybridization (BAC-FISH), we demonstrated that four of these deletions corresponded to fixed translocations (via homeologous exchanges). We were able to evaluate the size of the structural variations and their impact on the whole genome size, gene content, and allelic diversity. In addition, the evolution of fertility was assessed, to better understand the difficulty encountered by novel polyploid individuals before the putative formation of a novel stable species.

Polyploidy, or whole genome duplication (WGD), has played a major role in the evolutionary history of eukaryotes, especially in flowering plants (Soltis *et al.* 2014). Recent genomic analyses revealed that all angiosperms have been subjected to at least one round of polyploidy in their evolutionary history, and are thus considered paleopolyploids (Garsmeur *et al.* 2014). Despite the numerous studies performed on the origin and evolution of polyploids, it is still unknown how these species stabilized their genome and successfully established.

To determine the immediate impact of polyploidy on genome evolution, studies of either resynthesized or natural and recent allopolyploids (neoallopolyploids) have been performed. Contrasting results were observed, with some allopolyploid species presenting a rather unchanged genome (*i.e.*, *Spartina* and *Gossypium*), while others had highly shuffled genomes (*i.e.*, Arabidopsis, *Brassica napus*, or *Tragopogon*) (Liu *et al.* 2001; Baumel *et al.* 2002; Pontes *et al.* 2004; Mestiri *et al.* 2010; Szadkowski *et al.* 2010, 2011; Xiong *et al.* 2011; Chester *et al.* 2013, 2015). In plants, allopolyploidy can be at the origin of a genomic and transcriptomic shock, including gene conversion events, activation of transposable elements, chromatin remodeling, DNA methylation changes, and transcriptional or post-transcriptional changes (Madlung *et al.* 2002; Kashkush *et al.* 2003; Salmon *et al.* 2005, 2010; Gaeta *et al.* 2007; Marmagne *et al.* 2010; Szadkowski *et al.* 2010; Buggs *et al.* 2011). Overall, these various structural and functional changes increase the potential of polyploid species toward functional plasticity and evolutionary novelties, contributing to the phenotypic variability that may enable the species to exploit a wider range of environmental conditions (Pires *et al.* 2004; Fawcett *et al.* 2009; Van De Peer *et al.* 2009; Jiao *et al.* 2011).

A major challenge facing the successful establishment of a novel polyploid species reproducing sexually is its fertility. For several species, such as oilseed rape (*Brassica napus*), or wheat (*Triticum aestivum*), it has been established that the initial hybridization and its genome doubling are rare events. For that reason, the first few allopolyploid plants will produce seeds by self-fertilization to generate a founder population of the new species. The size and structure of this initial population in the first generations strongly depend on karyotype instability, and the level of aneuploid progenies produced (Zhang *et al.* 2013a). Recent studies examining resynthesized allopolyploids (allotetraploid *B. napus* and allohexaploid *Triticum aestivum*) or neoallopolyploids (allotetraploid *Tragopogon* species) have shown that novel allopolyploids may present aneuploid progenies (Xiong *et al.* 2011; Chester *et al.* 2013, 2015; Zhang *et al.* 2013b). Two different classes of aneuploids were considered: (i) compensated aneuploids (also called hidden aneuploids), with the expected number of chromosomes, but with a loss and gain of homeologous chromosomes; and (ii) numerical aneuploids, with an unexpected chromosome number. Relatively low levels of numerical aneuploids (∼10%) were observed in resynthesized allohexaploid wheat (selfed generation 2) (Zhang *et al.* 2013a), and in natural populations of neoallotetraploid *Tragopogon* species (∼40 generations old) (Chester *et al.* 2013, 2015) compared to previous observations (∼60%) in resynthesized *B. napus* populations (S5:6) obtained by controlled selfing (Xiong *et al.* 2011). In this latter species, a lower seed yield was observed in the numerical aneuploids compared with the compensated aneuploids (Xiong *et al.* 2011).

Oilseed rape (*Brassica napus* L., AACC, 2*n* = 38) is an excellent model species to study the immediate structural evolutionary dynamics of allopolyploid species, and the effect of natural selection on its evolution. *B. napus* is a recent, partially allogamous allopolyploid species that formed ∼7500 years ago (Chalhoub *et al.* 2014) after the hybridization and genome doubling of two closely related diploid species, *B. rapa* (AA, 2*n* = 20) and *B. oleracea* (CC, 2*n* = 18) (Nagaharu 1935; Inaba and Nishio 2002). This species was presumably selected by humans because a wild population of *B. napus* was not discovered. To date, it remains unclear how selection allowed genome stabilization. In resynthesized *B. napus* populations, genetic studies of A01/C01 chromosomes using molecular markers have revealed that the first meiosis acts as a “genome blender” because 50% of the gametes presented homeologous exchanges between these two chromosomes (Szadkowski *et al.* 2010). The polyploid formation pathway (somatic doubling *vs.* unreduced gametes) impacts the size and number of translocations (Szadkowski *et al.* 2011). In addition, genetic and cytogenetic analyses of single seed descent obtained by self-fertilizing of independent synthetic *B. napus* lines deriving from an initial hybridization of *B. oleracea* “TO1000” and *B. rapa* “IMB218” revealed that these lines presented numerous nonreciprocal translocation events (Gaeta *et al.* 2007; Xiong *et al.* 2011), and a high level of numerical aneuploids (∼60%) (Xiong *et al.* 2011). This latter study was performed using resynthesized *B. napus* allopolyploids obtained by controlled self-fertilization, regardless of the genome stability, pollen viability, and seed set, thus permitting the study of highly unstable genomes with a very low reproductive fitness. However, it is most likely that individuals from the first *B. napus* population were produced via open pollination to allow outcrossing. To date, the effects of open pollination on the structural evolutionary dynamics, meiotic behavior, and fertility of resynthesized allotetraploid *B. napus* are unknown. In addition, to our knowledge, the size (in megabases) of the structural variations (SVs) in resynthesized *B. napus*, and their impact on gene content and allelic diversity, have been analyzed in only one synthetic (Chalhoub *et al.* 2014), precluding the establishment of a general trend. To better understand the role of the reproductive mode on karyotype stability following allotetraploidization in *B. napus*, and before a putative speciation event, we created two different resynthesized *B. napus* populations obtained either by controlled selfing or open pollination, with the latter revealing a higher fertility. We then conducted a detailed analysis of the populations obtained from open pollination by investigating their aneuploidy level, meiotic behavior, and genome-wide SVs. From these analyses, we observed that the resynthesized *B. napus* populations presented a very low level of numerical aneuploids. The genetic (using a 60K Illumina array) and cytogenetic studies of 33 G3 individuals obtained by open pollination allowed the detection of unstable meiosis and extensive shuffling of the genome of these plants. BAC-FISH experiments were subsequently performed to validate four of the SVs identified using the array, allowing us to determine that these deletions of genomic regions resulted from translocations that were fixed at the homozygous stage (via homeologous exchanges, hereafter referred to as HE). We were also able to determine the size of these SVs, and their impacts on gene content and allelic diversity. Overall, our results shed light on the impact of open pollination on the genome dynamics, fertility, and putative successful establishment of novel allopolyploids.

## Materials and Methods

### Plant material

**Figure 1:**
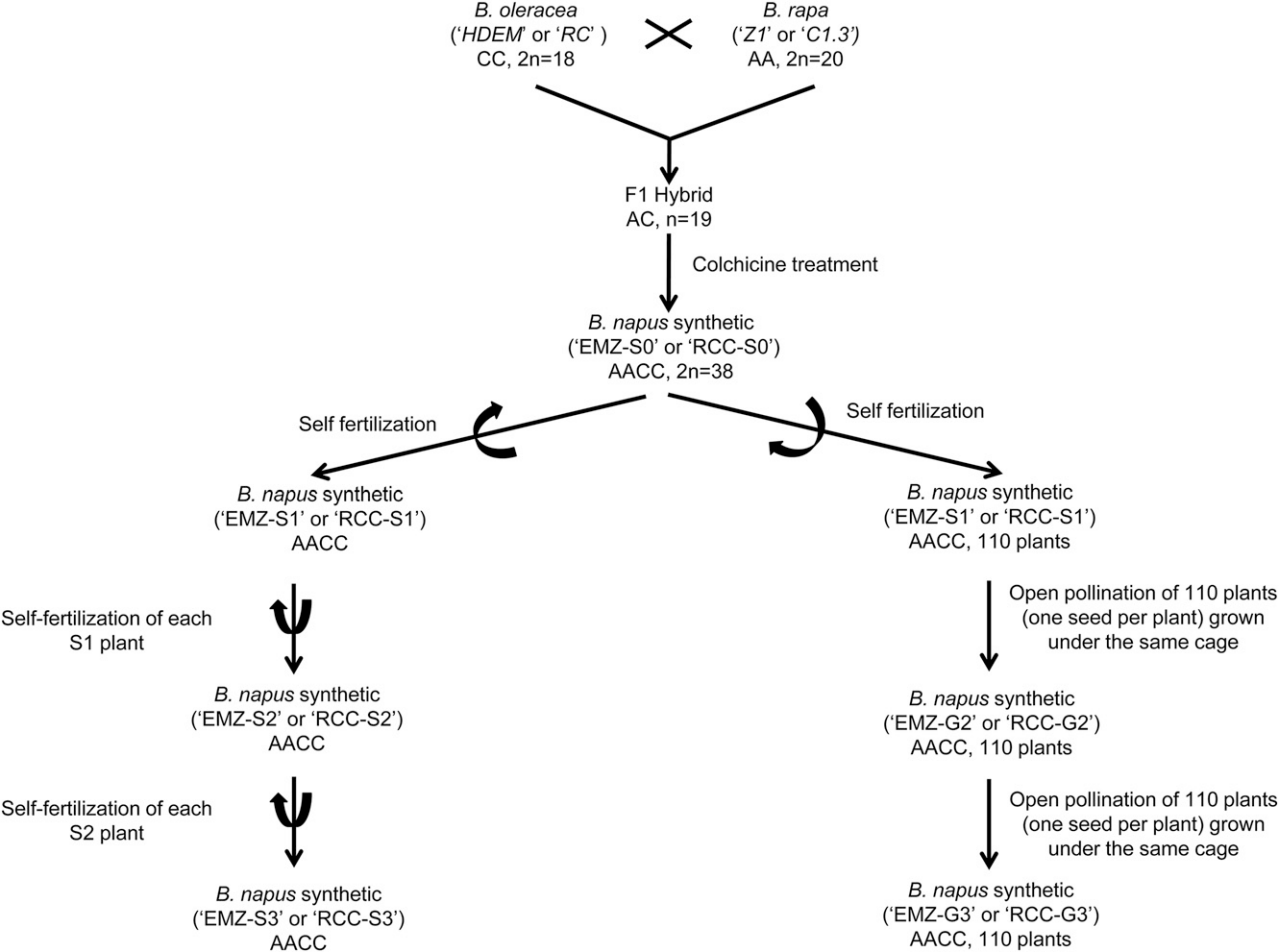
Schematic representing how the resynthesized *B. napus* populations used in this study were created. The names of the different *B. oleracea* and *B. rapa* varieties used to create these populations are indicated. The method (self-fertilization or open pollination) used to obtain the different generations is mentioned.

Two different resynthesized *B. napus*, named “RCC” and “EMZ,” were created following the experimental design detailed below and presented in Figure 1. First, two crosses between a *B. oleracea* and a *B. rapa* individual were performed. The first cross was performed between a *B. oleracea* var. alboglabra, a doubled haploid line “RC34” (mother plant) with a *B. rapa* plant “C1.3” belonging to a fodder variety named “chicon” var. rapifera. Similarly, a cross between two fully homozygous doubled haploid lines was performed: *B. oleracea* var. *botrytis italica* “HDEM” (2*n* = 2*x* = 18, mother plant) with *B. rapa* var. trilocularis “Z1” (2*n* = 2*x* = 20). All these genotypes were provided by the Biological Resource Center (CRB BrACySol, Rennes-Ploudaniel, France) except Z1 (provided by K. C. Falk, Agriculture and Agri-Food Canada, Ottawa, ON). The resulting amphiploid hybrids (AC, 2*n* = 2*x* = 19) were somatically doubled using colchicine (Chevre *et al.* 1989), leading to one RCC-S0 (2*n* = 4*x* = 38) and EMZ-S0 (2*n* = 4*x* = 38)-resynthesized *B. napus* individual from the first and second cross, respectively. The meiotic behavior and chromosome number of the F1 and S0 plants (2*n* = 38) were evaluated (Szadkowski *et al.* 2010, 2011). By selfing (the hand pollination of floral buds before anthesis) one RCC-S0 and one EMZ-S0 plant, we produced RCC-S1 and EMZ-S1 progenies. Thereafter, two strategies (for each genetic background) were used to produce the subsequent generations. In both cases, one seed per plant was used to produce the progeny (Single Seed Descent method). In the first strategy, manual self-fertilization of 11–18 S1 plants was performed to produce S2 and S3 progenies. In the second strategy, 110 S1 plants were grown under the same cage (one cage for RCC and one for EMZ), and in the presence of flies to ensure open pollination, and to facilitate outcrossing between plants. The following year, one seed per 110 G2 plants (from either RCC or EMZ) was grown in a cage (with flies) to obtain the G3 progenies (these plants are hereafter referred as “G3”). As a control of seed fertility for *B. napus* varieties, 50 plants of the spring variety Drakkar (provided by the CRB BrACySol, Rennes-Ploudaniel, France) were grown each year in another cage, but under the same conditions used for the populations obtained from open pollination.

### DNA extraction and SNP genotyping

Genomic DNA of 18 RCC-G3 and 15 EMZ-G3 plants obtained from outcrossing (all presenting 38 chromosomes), as well as of the diploid parents (“RC34,” “C1.3,” “HDEM” and “Z1”), and the resynthesized S0 *B. napus* (“EMZ-S0” and “RCC-S0”), were extracted from young leaves using a Qiagen kit. For all these plants, genotyping was performed using the Illumina (http://www.illumina.com/) Brassica 60K Infinium SNP array (Clarke *et al.* 2016). Hybridizations were performed according to the standard procedures provided by the manufacturer for each genomic DNA extracted. Three technical replicates were realized for each diploid parent and each resynthesized S0 *B. napus*.

### Data analyses

The genotyping data from the Illumina *Brassica* 60K array were visualized using Genome Studio V2011.1 (Illumina, Inc., San Diego, CA), and processed with a manually adapted cluster file. The positions on the *B. napus* chromosomes of the SNP markers present on the array were obtained by blasting the 52,157 sequence contexts against the *B. napus* cv. Darmor reference genome sequence assembly (version 4.1: Chalhoub *et al.* 2014). Only the BLAST hits with a minimum of 90% global overlap and 90% identity were retained. Subsequently, only markers presenting no more than one BLAST hit on each subgenome were kept, enabling SNPs with the potential to hybridize at paralogous regions to be discarded.

### Identification of structural rearrangements

To identify putative structural rearrangements in our G3 populations from resynthesized S0 *B. napus*, the markers that were homozygous and polymorphic (AA *vs.* BB) between the diploid parents and heterozygous (AB) in the S0 of each population were first determined. In this step, only the markers, for which identical genotype data were obtained for all technical replicates, were considered. Thereafter, a custom python script was applied to identify in each G3 plant, which of the polymorphic markers previously retained had lost one parental allele. The positions on the *B. napus* chromosomes of markers showing either additivity (both parental alleles) or loss of one parental allele were graphically represented for each G3 *B. napus* plant using CIRCOS (Krzywinski *et al.* 2009). By determining whether consecutive markers displayed the loss of one parental allele (from the same parent), we were able to identify large deletions and infer the size and position of each deleted region. The presence of an active centromere in the deleted region was then determined using the positions of the *B. napus* centromeres (Mason *et al.* 2016). In addition, we also determined whether deletions were present in the distal region of a chromosome arm: we considered the last 30% of each chromosome arm as the distal region.

### Statistical analyses

To determine whether structural rearrangements were statistically prevalent on a specific *B. napus* subgenome, the proportion of each deleted region in each RCC-G3 and EMZ-G3 individual was estimated based on its subgenome size. Because the data did not follow a normal distribution, the nonparametric Wilcoxon rank-sum test (Wilcoxon 1945) was applied independently for RCC-G3 and EMZ-G3 plants.

Similarly, the proportion of each deleted region in each RCC-G3 and EMZ-G3 was estimated per chromosome to determine whether each *B. napus* chromosome was equivalently subjected to structural rearrangements. Due to the non-normality of the data, the nonparametric Friedman rank-sum test (Friedman 1937) was applied. For all these analyses, only *p*-values <0.05 were considered significant.

Similar statistical analyses were performed by considering the proportion of genes that were deleted in a subgenome and per chromosome.

### Validation and identification of the underlying mechanism using BAC-FISH

To validate some of the structural rearrangements identified using the SNP array, and to identify the mechanisms involved (*i.e.*, deletion *vs.* translocation after HE), BAC-FISH combined with GISH-like experiments were performed for plants showing major structural modifications. In these plants, as well as in their diploid parents, chromosome preparations were performed according to procedures detailed in Ksiazczyk *et al.* (2011). For the BAC-FISH experiments, we identified BACs from *B. rapa* (Mun *et al.* 2008) and *B. napus* (Chalhoub *et al.* 2014) libraries that were present in the rearranged regions of some G3 individuals. Some of these BACs had previously been used in similar cytogenetic experiments (Xiong and Pires 2011), whereas others were identified in this study by developing primer pairs specific to the deleted region in a synthetic individual, and screening the *B. napus* BAC library (Chalhoub *et al.* 2004). All four BACs used in this study hybridized to one pair of chromosomes in each diploid species, and to two homeologous chromosome pairs (four signals) in the *B. napus* Darmor variety (see the positions of each of these BACs used for the BAC-FISH experiment in Supplemental Material, Table S1). For example, clone KBrB086G22 hybridizes to A02 chromosomes in *B. rapa* (two signals), C02 chromosomes in *B. oleracea* (two signals), and both A02 and C02 chromosomes in *B. napus* (four signals) (Xiong and Pires 2011). Thereafter, to identify the A or C chromosome to which each BAC hybridized in an allotetraploid *B. napus*, we used, on the same slide, the Bob014O06 BAC clone that hybridizes to C-genome chromosomes in *B. napus* (GISH-like) (Szadkowski *et al.* 2010). Each BAC present within a deleted region of a synthetic was labeled by random priming with biotin-14-dUTP (Invitrogen, Life Technologies), whereas the Bob014O06 BAC clone was labeled by random priming with Alexa 488-5-dUTP.

Biotinylated probes were immunodetected using Texas Red avidin DCS (Vector Laboratories), and the signal was amplified with biotinylated anti-avidin D (Vector Laboratories). The chromosomes were mounted and counterstained in Vectashield (Vector Laboratories) containing 2.5 µg/ml 4′,6-diamidino-2-phenylindole (DAPI). Fluorescence images were captured using a CoolSnap HQ camera (Photometrics, Tucson, Ariz) on an Axioplan 2 microscope (Zeiss, Oberkochen, Germany), and analyzed using MetaVue (Universal Imaging Corporation, Downingtown, PA).

### Meiotic behavior

The flower buds of only G3 plants obtained by open pollination (109 plants) were harvested, enabling the characterization of their meiotic behavior and the establishment of their chromosome number following the protocol described by Suay *et al.* (2014). Per plant, ∼20 pollen mother cells (PMCs) in metaphase I of meiosis were analyzed.

### Assessment of fertility in resynthesized *B. napus* populations

To assess the fertility of the resynthesized *B. napus* plants (S0), the number of seeds per pollinated flower was assessed for S1, S2, and S3 plants obtained by controlled manual selfing. For the G1, G2, and G3 plants growing under cages, as well as for the *B. napus* var. Drakkar, the number of seeds per pollinated flower and per pod was assessed for each plant by counting the number of pods per 50 flowers on the first floral hampers, and the number of seeds per 50 pods.

### Data availability

The positions on the *B. napus* chromosomes of each BAC used in the BAC-FISH experiments are shown in Table S1. The positions on the *B. napus* chromosomes of the different deletions observed in each synthetic *B. napus* individual investigated in this study are shown in Table S2. Finally, Table S3 contains the list of the primer pairs designed (names, sequences, and positions on *B. napus* chromosomes) to identify the presence of the putative genic conversion in some synthetic *B. napus* individuals.

## Results

### Impact of open pollination *vs.* selfing on fertility

**Figure 2:**
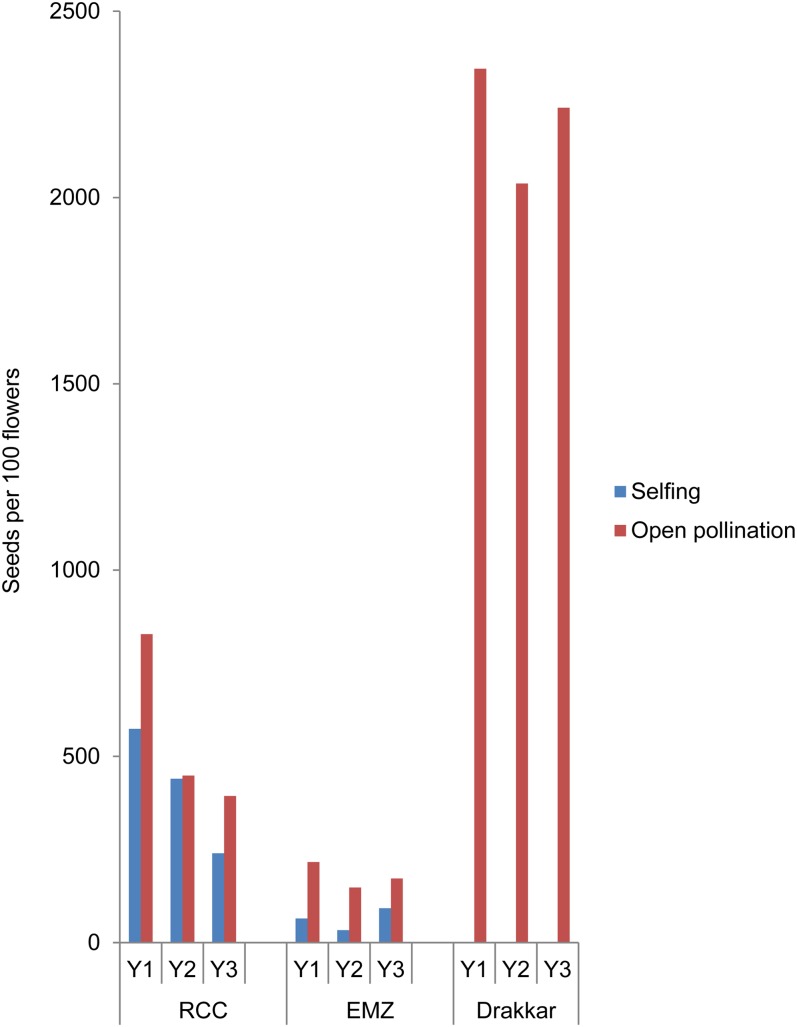
Fertility of EMZ and RCC resynthesized *B. napus* populations obtained by either self-fertilization or open pollination, compared to the *B. napus* Drakkar variety. The fertility, corresponding to the number of seeds per 100 flowers (*y*-axis), was assessed for each population for three consecutive generations (Y1, Y2, and Y3). Each year, the fertility of the Drakkar variety was also determined by growing 50 Drakkar plants using the same growth conditions described for the resynthesized populations produced by open pollination.

The evolution of fertility was evaluated in both “RCC” and “EMZ” progenies, and compared with the Drakkar variety. In all cases, the fertility of the resynthesized *B. napus* populations, which was assessed based on the number of seeds per 100 flowers, was drastically reduced compared with Drakkar (3- and 10-fold reduction in RCC-G1 and EMZ-G1, respectively) (Figure 2). We also observed that the fertility was significantly higher (*t*-test, *p* < 0.05) for plants obtained by open pollination except at the G2 generation for RCC, where no significant difference was observed (*t*-test, *p* = 0.815) (Figure 2). In all populations, fertility significantly decreased over subsequent generations (*t*-test, *p* < 0.05), except in self-fertilized EMZ plants. In the latter case, the lack of a decrease in fertility was due to the extremely low fertility of these plants since the first generation (Figure 2).

### Chromosome number and meiotic behavior of G3 plants obtained via open pollination of resynthesized B. napus

The chromosome number, and meiotic behavior at metaphase I, of 109 RCC-G3 and EMZ-G3 individuals, as well as of the *B. napus* Drakkar variety, were determined. Most RCC (91%) and EMZ (95%) plants had 38 chromosomes, apart from a few numerical aneuploid plants that consistently presented one or two supernumerary chromosomes (*i.e.*, 39 or 40 chromosomes). Meiosis was evaluated in 15–18 randomly chosen G3 plants obtained from open pollination (per population). Meiosis was affected in these plants, all of which presented 38 chromosomes, with only 30–70% of the PMCs having 19 bivalents, compared to 100% in the *B. napus* Drakkar variety (Table 1). All G3 individuals showed meiotic instability, and presented multivalents and/or univalents. We observed at least one quadrivalent in 4–48% PMCs of all the plants genotyped (apart from RCC-G3-111), and one univalent (from 1 to 6 per cell) in 5–70% of PMCs.

**Table 1:** The chromosome number, meiotic behavior at metaphase I, and fertility of S0 and G3 resynthesized *B. napus*

			Average No. (Total No. Counted)							
Individual	No. of Chromosomes	No. of PMCs	Univalents	Bivalents	Trivalents	Quadrivalents	Pentav.	Percentage of PMCs with 19 Bivalents	Percentage of PMCs with Multivalents	Seed No. Per 100 Flowers
RCC-S0	38	25	0.08 (0–2)	18.64 (17–19)	0	0.16 (0–1)	0	80	16	177
RCC-G3-050	38	20	0.65 (0–2)	18.25 (17–19)	0.15 (0–1)	0.1 (0–1)	0	50	25	126
RCC-G3-051	38	21	0.38 (0–2)	18.24 (17–19)	0.19 (0–1)	0.14 (0–1)	0	57.14	33.33	195
RCC-G3-053	38	22	0.68 (0–4)	18.41 (17–19)	0.05 (0–1)	0.09 (0–1)	0	59.1	13.64	113
RCC-G3-054	38	20	0.7 (0–2)	18.1 (17–19)	0.1 (0–1)	0.2 (0–1)	0	40	30	84
RCC-G3-055	38	21	0.57 (0–4)	18.33 (17–19)	0	0.19 (0–1)	0	57.14	19	59
RCC-G3-056	38	20	1.15 (0–4)	18.15 (16–19)	0.05 (0–1)	0.1 (0–1)	0	50	15	103
RCC-G3-057	38	20	1.9 (0–6)	17.45 (15–19)	0.2 (0–1)	0.15 (0–1)	0	30	35	41
RCC-G3-059	38	20	0.4 (0–2)	18.6 (17–19)	0	0.1 (0–1)	0	70	10	196
RCC-G3-071	38	21	0.33 (0–4)	17.81 (15–19)	0	0.52 (0–2)	0	42.86	47.61	178
RCC-G3-072	38	15	0.67 (0–2)	18.53 (17–19)	0	0.7 (0–1)	0	60	6.66	214
RCC-G3-073	38	20	0.65 (0–2)	18.05 (16–19)	0.2 (0–1)	0.1 (0–1)	0.05 (0–1)	45	35	80
RCC-G3-074	38	20	0.5 (0–2)	18.15 (16–19)	0	0.3 (0–1)	0	50	30	51
RCC-G3-075	38	23	0.26 (0–2)	18.22 (17–19)	0.09 (0–1)	0.26 (0–1)	0	56.52	35	180
RCC-G3-076	38	20	0.4 (0–2)	18.3 (15–19)	0	0.25 (0–2)	0	60	25	180
RCC-G3-077	38	21	0.29 (0–2)	18.48 (17–19)	0	0.19 (0–1)	0	66.67	19.05	226
RCC-G3-111	38	20	1.2 (0–4)	18.25 (16–19)	0.1 (0–1)	0	0	50	10	317
EMZ-S0	38	16	0.13 (0–2)	18.31 (17–19)	0	0.31 (0–1)	0	62.5	31.25	98
EMZ-G3-078	38	20	0.6 (0–2)	17.95 (15–19)	0.1 (0–1)	0.3 (0–2)	0	45	35	1.88
EMZ-G3-082	38	20	0.4 (0–2)	18.2 (16–19)	0	0.3 (0–1)	0	55	30	3.12
EMZ-G3-084	38	20	0.55 (0–2)	18.25 (17–19)	0.25 (0–1)	0.05 (0–1)	0	55	30	0.50
EMZ-G3-086	38	21	0.86 (0–4)	18.29 (17–19)	0	0.14 (0–1)	0	47.62	14.28	Not available
EMZ-G3-088	38	20	0.65 (0–2)	18.25 (17–19)	0.15 (0–1)	0.1 (0–1)	0	50	25	0.00
EMZ-G3-090	38	21	0.38 (0–4)	18.14 (17–19)	0	0.33 (0–1)	0	52.38	33.33	5.17
EMZ-G3-092	38	20	0.2 (0–2)	18.2 (17–19)	0	0.35 (0–1)	0	55	35	2.50
EMZ-G3-094	38	20	0.3 (0–2)	18.05 (17–19)	0	0.4 (0–1)	0	45	40	25.93
EMZ-G3-098	38	20	0.1 (0–2)	18.25 (16–19)	0	0.35 (0–1)	0	65	35	8.82
EMZ-G3-100	38	22	0.9 (0–4)	17.82 (15–19)	0	0.36 (0–2)	0	36.36	31.82	1.76
EMZ-G3-104	38	20	0.45 (0–2)	18.4 (17–19)	0.05 (0–1)	0.15 (0–1)	0	60	20	5.96
EMZ-G3-106	38	25	1. 04 (0–4)	18.6 (16–19)	0.16 (0–1)	0.04 (0–1)	0	44	20	0.00
EMZ-G3-108	38	21	0.24 (0–2)	18.09 (17–19)	0.14 (0–1)	0.29 (0–1)	0	52.38	42.8	0.00
Drakkar var.	38	20	0	19	0	0	0	100	0	1079.56

### Genotyping data obtained from the 60K array, and the positions of the SNP markers on the B. napus chromosomes

Genotyping data were obtained using the Brassica 60K SNP array for 18 RCC-G3 plants and 15 EMZ-G3 (2*n* = 4*x* = 38), as well as for their parental diploid parents (“HDEM,” “Z1,” “RC,” and C1.3), and the two initial allotetraploid resynthesized S0 plants (“RCC-S0” and “EMZ-S0”). From this array, composed of 52,157 SNP markers, 15,058 hybridized in both *B. rapa* and *B. oleracea* (in either “HDEM” or “RC34” for *B. oleracea*, and in either “Z1” or “C1.3” for *B. rapa*), and thus hybridized to both homeologous chromosomes in our resynthesized *B. napus*. Using the recently released *B. napus* cv. Darmor genome sequence (Chalhoub *et al.* 2014), we identified the positions of all the markers on the *B. napus* chromosomes. A total of 17,115 markers were specific to the C-subgenome, 12,969 were specific the A-subgenome, and 9504 hybridized to both the A- and C-subgenomes (homeologs).

The positions of these different SNP markers on *B. napus* chromosomes are presented in Figure S1. Using these stringent filtering BLAST parameters, we observed several BLAST hits on the same subgenome (paralogs) for <8% of the markers, which were thus discarded from further analyses. Indeed, 534, 573, and 346 heterozygous markers were observed in the “HDEM,” “RC34,” and “Z1” DH lines, indicating that a low percentage of the SNP markers present on the array may hybridize to paralogous regions, as previously mentioned (Clarke *et al.* 2016).

### Genotyping and identification of putative structural rearrangements in resynthesized B. napus populations

**Figure 3:**
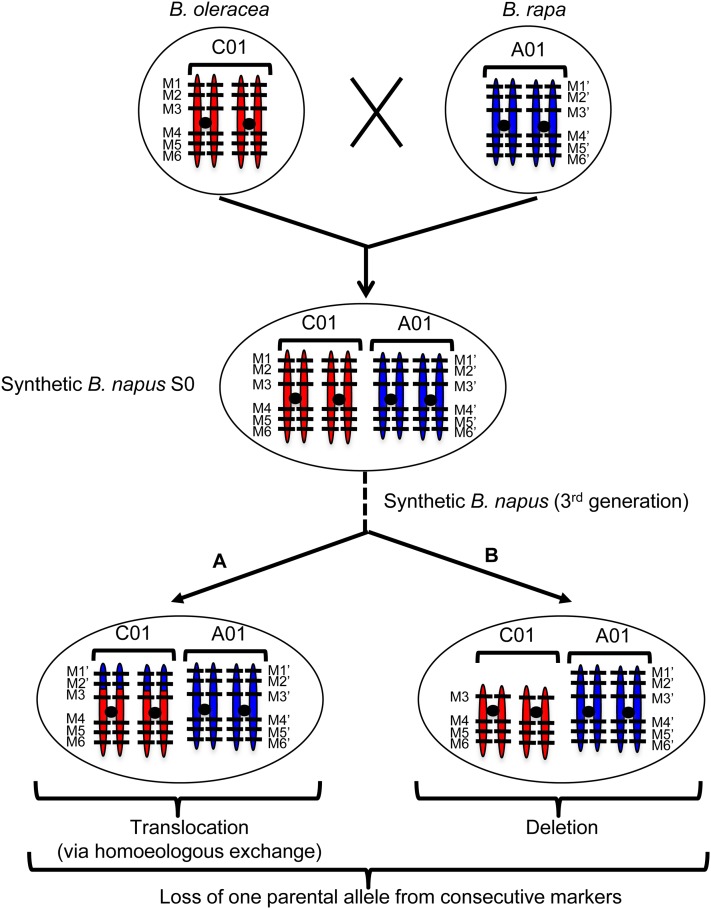
Methodology used to analyze the SNP genotyping data obtained from the Illumina *Brassica* 60K array and identification of structural rearrangements in each of the *B. napus* synthetic lines (third generation). To identify putative structural rearrangements in each *B. napus* synthetic line (G3), only markers that were polymorphic between *B. rapa* and *B. oleracea* (*i.e.*, “HDEM” and “Z1,” or “RC34” and “C1.3”), and heterozygous in the first allotetraploid synthetic individual created (“EMZ-S0” or “RCC-S0”) were considered. The position of these markers was then inferred using the *B. napus* cv. Darmor reference genome sequence assembly (version 4.1, Chalhoub *et al.* (2014)). This graphical representation shows an example of six markers that were polymorphic in the two diploid parental lines, which were present on C01 in *B. oleracea* (M1–M6), on A01 in *B. rapa* (M1′–M6′), and on the A01/C01 homeologous chromosomes (heterozygous markers) in *B. napus* S0. The loss of one parental allele from two consecutive markers (M1 and M2 on C01 herein) in a synthetic *B. napus* individual indicates the presence of a structural rearrangement resulting from either (A) a translocation (via homeologous exchange) or (B) a deletion. For these two cases, the reciprocal situation may also be observed [*i.e.*, duplication of a C01 region (A) or deletion of an A01 region (B)].

**Figure 4:**
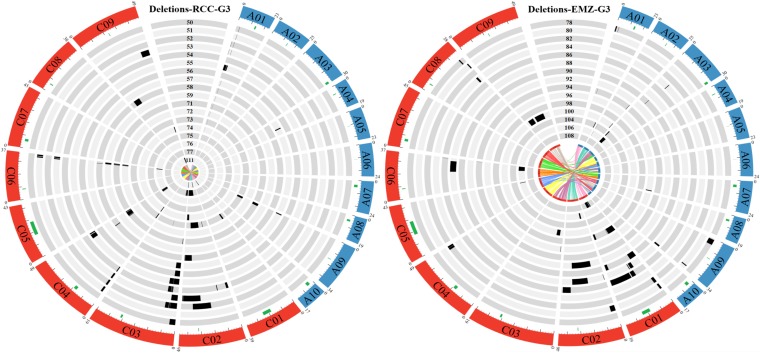
Graphical representation of the deletions identified in each “RCC” (left) or “EMZ” (right) G3 plant. The *B. napus* chromosomes belonging to the A and C subgenomes are shown in blue and red, respectively. The size of each chromosome (in megabases) is indicated above each chromosome, and a ruler is drawn underneath each chromosome, with larger and smaller tick marks every 10 and 2 Mb, respectively. The locations of active centromeres (Mason *et al.* 2016) are indicated by green rectangles under each chromosome. Each circle represents one G3 plant. For each individual, a black rectangle represents a deleted region. The last inner circle corresponds to the homeologous relationship between the A and C chromosomes.

To identify SVs in each G3 individual from resynthesized *B. napus*, only the 60K SNP markers that hybridized to both diploid progenitors, and were polymorphic between them, were considered. For such markers, we verified that they were also heterozygous in resynthesized S0 *B. napus*. In total, 1757 markers were polymorphic between HDEM (*B. oleracea*) and Z1 (*B. rapa*), and heterozygous in EMZ-S0. A total of 2405 markers were polymorphic between RC (*B. oleracea*) and C1.3 (*B. rapa*), and heterozygous in RCC-S0, and most of these markers could be localized on *B. napus* chromosomes (Figure S2 and Figure S3). Overall, there was an average of one polymorphic marker every 243 kb for the RCxC1.3 (RCC) combination, and 316 kb for the HDEMxZ1 (EMZ) combination. These markers covered all *B. napus* chromosomes but with a lower density in the pericentromeric compared with distal regions. The lowest and highest marker densities were observed for the C01 (one every 452 kb), and the A03 (one every 108 kb) chromosomes for the RCC plants. For the EMZ plants, the lowest and highest marker density corresponded to C09 (one every 808 kb) and A08 (one every 153 kb). Using these polymorphic markers alone, we subsequently assessed whether one parental allele was missing from each of the 18 RCC-G3, and the 15 EMZ-G3 individuals obtained via open pollination (an example is provided for EMZ-G3-078 in Figure S4). In the RCC-G3 population, 0 (RCC-G3-075) to 245 SNP markers (RCC-G3-053 and RCC-G3-055) presented a loss of one of the two parental alleles, whereas between 12 (EMZ-G3-096) and 160 SNPs (EMZ-G3-088) demonstrated such a loss in the EMZ population. To identify losses of large genomic regions in an individual, we next determined whether consecutive markers revealed a loss of one parental allele (from the same parent) (Figure 3). Using this method, we identified between one and six large (mean: 3.06), and between zero and seven deleted regions (mean: 3.78) in RCC and EMZ individuals, respectively (Figure 4 and Table S2). In each population, few identical deletions (same borders) were present in several individuals, most likely resulting from the same initial rearrangements during the first meiosis. When considering the presence of identical deletions in several individuals resulting from a single event, a mean of 1.83 and 2.73 structural rearrangements were observed per RCC and EMZ individual, respectively (Figure 4).

The size of each deleted region was evaluated (Table S2), and ranged from 0.138 Mb (0.59% of A01) to 24.78 Mb (53.6% of C02: RCC-G3-077) in RCC-G3 plants, and from 0.048 Mb (0.12% of C01: EMZ-G3-092 and EMZ-G3-106) to 23.36 Mb (50.55% of C02: EMZ-G3-092) in EMZ G3 plants. Most of these deletions were in the distal region of a chromosome arm (79.41% in RCC and 67.4% in EMZ), and a few contained active centromeres (2.94% in RCC and 8.7% in EMZ) of the C01, C02, or C09 chromosomes. When cumulating the size of all deletions observed in the genome of a G3 plant, the individuals presenting the least and most important rearranged genomes corresponded to EMZ 072 (2.43 Mb: 0.37% of the Darmor genome) and EMZ 077 (35.00 Mb: 5.42% of the Darmor genome). Interestingly, no loss of any parental allele was identified for RCC-G3-075, despite the presence of 19 bivalents in only 57% of its PMCs (Table 1).

**Figure 5:**
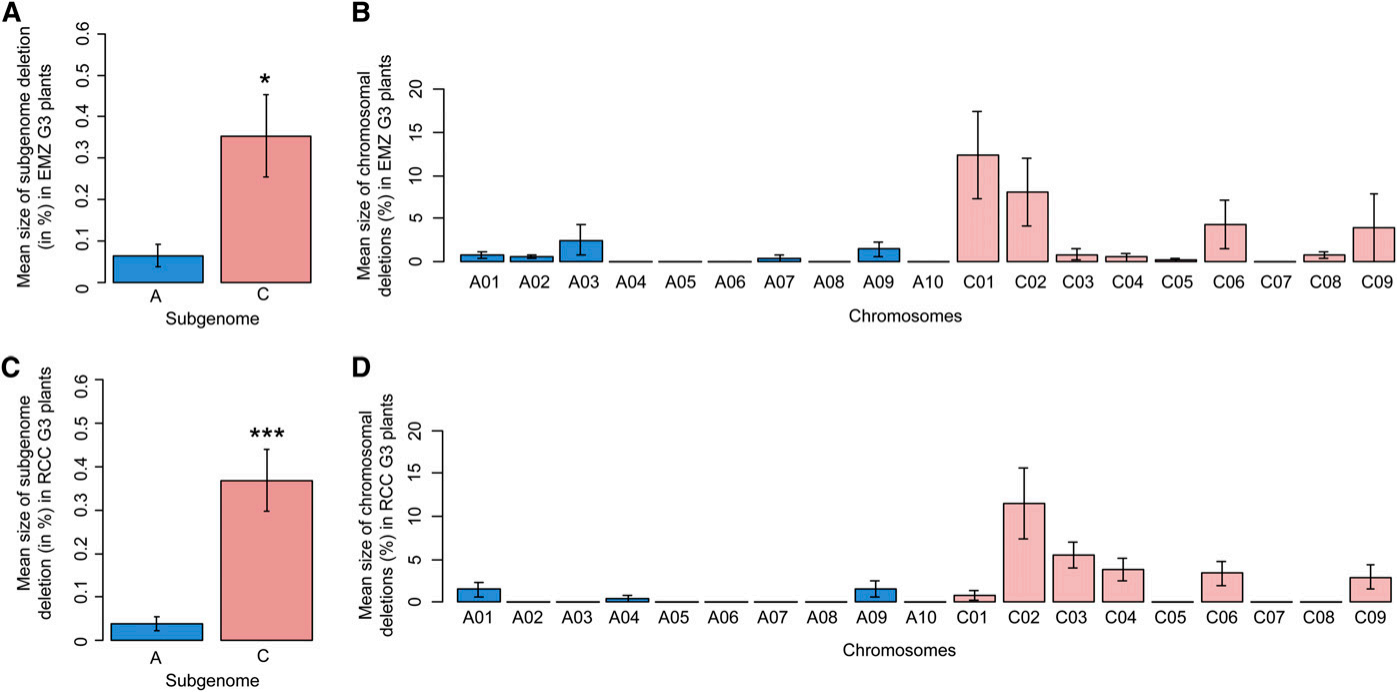
Mean size of subgenome deletions (in megabases) in EMZ (A) or RCC G3 plants (C). The mean size of deletions per chromosome (in megabases) for EMZ (B) or RCC plants (D) is also presented. The presence of significant differences using the Wilcoxon rank-sum test (Wilcoxon 1945) is indicated by a star(s).

To determine whether a subgenome was more prone to deletions, the total size of the deleted regions observed for each subgenome in a G3 population was determined. Subsequently, the proportion of deleted regions in a subgenome was calculated by taking into account the relative size of the A and C subgenome in *B. napus*. These analyses revealed significantly more deleted regions (total size) from the C than the A subgenome in both RCC (Wilcoxon rank-sum test, *W* = 12,058, *p*-value = 3.49e−05), and EMZ (Wilcoxon rank-sum test, *W* = 9123.5, *p*-value = 0.01823) (Figure 5). In addition, we observed that a larger number of C than A chromosomes were subjected to deletions. Indeed, the proportion of the deleted region per chromosome was not homogenous in RCC (Friedman rank-sum test, chi-squared = 133.5809, d.f. = 18, *p*-value < 2.2e−16), or EMZ (Friedman rank-sum test, chi-squared = 52.1907, d.f. = 18, *p*-value = 3.497e−05). Six and eight of the nine C chromosomes had a deleted fragment in the RCC and EMZ G3 populations, respectively, compared with three and five A chromosomes for RCC and EMZ. Deleted regions were never observed in the C07, A05, A06, A08, and A10 chromosomes in either of the genotyped RCC or EMZ-G3 individuals. Conversely, they were present in the C01, C02, C06, C09, A03, and A09 chromosomes of both populations, especially C02, C06, and C09. The C01 chromosome had the largest proportion of deleted regions in EMZ but not in RCC (Figure 5).

### Identification of translocations (via HE) in resynthesized B. napus populations

**Figure 6:**
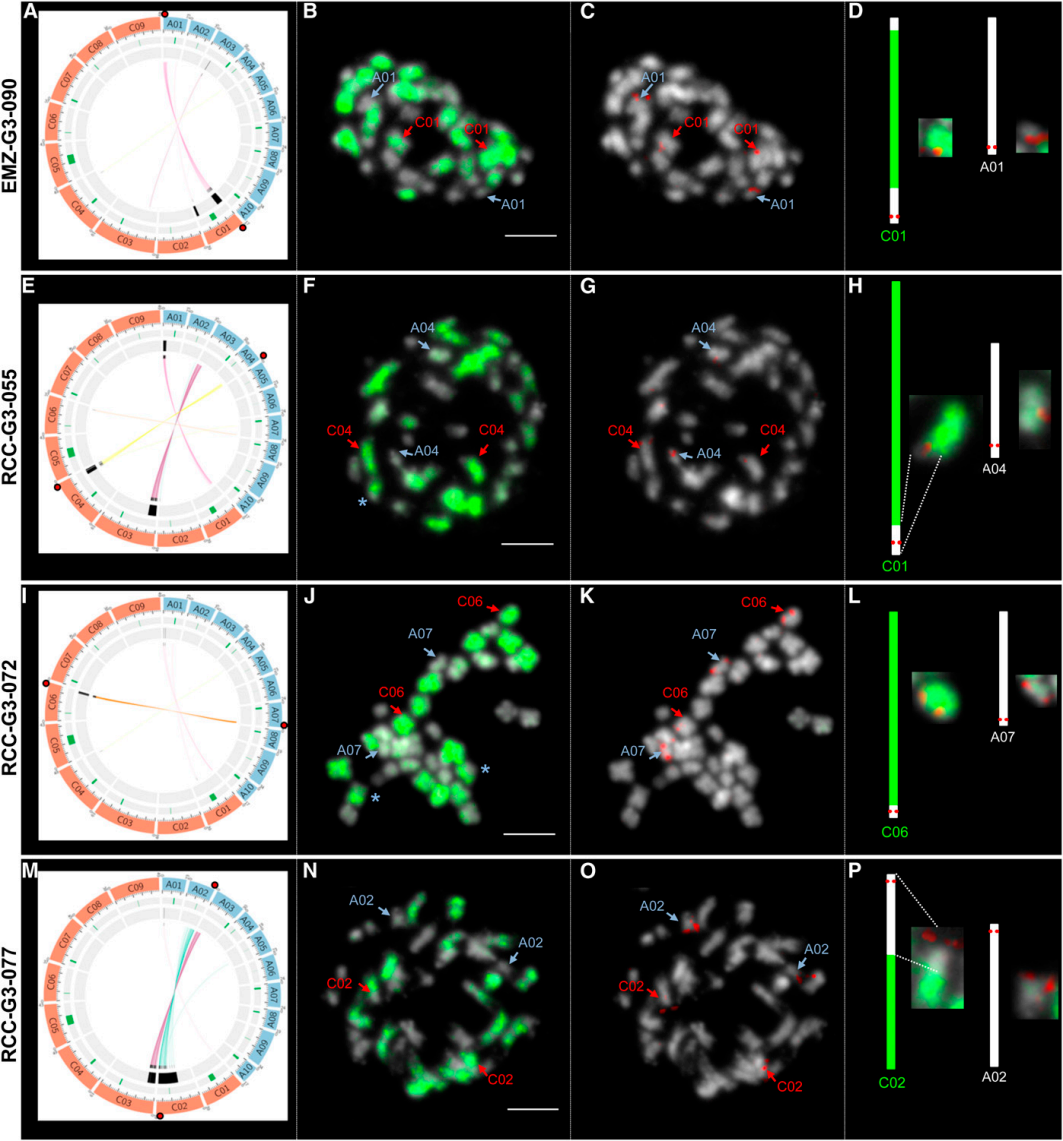
Identification of translocations (via homeologous exchanges) in four G3 plants using BAC-FISH. BAC-FISH was performed on somatic metaphase chromosomes with a BAC within a deleted region (red), and the Bob014O06 clone that identifies all C chromosomes (green). Black boxes indicate the positions of the deleted regions identified in these four G3 plants, whereas green boxes show the position of the centromeres (A, E, L, and M). The links in the most inner circle of the circos representations indicate the homeologous relationships of the markers presenting the loss of one parental allele in the synthetic plant (A, E, L, and M). The positions of the BACs (specific to a pair homeologous chromosomes) used to validate the presence of a structural rearrangement in each synthetic *B. napus* are represented by red dots (A, E, L, and M). The identification of a translocation between A01 and C01 in EMZ-G3-090 using Bna191G20 (in red) and Bob014O06 (in green) clones (A–D); between A04 and C04 in RCC-G3-055 using KBrH009I04 (in red), and Bob014O06 (in green) clones (E–H); between A07 and C06 in RCC-G3-072 using KBrB021P15 (in red) and Bob014O06 (in green) clones (I–L); and between A02 and C02 in RCC-G3-077 using KBrB086G22 (in red) and Bob014O06 (in green) clones (M–P). Additional translocations between the A and C chromosomes are indicated by a star (*). Chromosomes were counterstained with DAPI (blue). Bars represent 5 µm. A schematic representation of the expected structure of the translocated chromosomes (drawn to scale) is presented (D, H, L, and P).

The deletions (lost alleles) identified for each G3 plant may be the result of either a deletion or a translocation (via HE) that was fixed at the homozygous stage. To identify the type of rearrangement involved, we first examined the signal intensity of the SNP markers. Indeed, in the event of a translocation (after an HE), the signal intensity of the markers that showed a loss of one parental allele was hypothesized to be doubled for the other homeologous parental allele (Figure 3). To test this hypothesis, the relative copy number of such markers was estimated from the log 2 ratio of the signals observed in the individual that had lost a parental allele and the other diploid parent (data not shown). In addition, to evaluate the fluctuation in the hybridization signal for SNP markers presenting the same dosage, dominant markers (*i.e.*, present in one diploid parent but absent in the other) were also used. No significant increase in copy number could be observed in any of the rearranged regions, suggesting that our data were not adapted to evaluate the allele dosage. Consequently, to unambiguously determine the type of rearrangement involved in these chromosome fragment losses, we performed BAC-FISH experiments using BAC probes from a deleted region of a G3 plant, and the Bob014O06 BAC clone (Howell *et al.* 2002) that specifically hybridizes to all C chromosomes in *B. napus* (Szadkowski *et al.* 2010; Suay *et al.* 2014). Due to the high sequence similarity between the A and C subgenomes, the BACs specific to a deleted region also consistently hybridized to the homeologous chromosome in *B. napus* cv. Darmor, and thus provided four signals (Xiong and Pires 2011). Using a BAC clone present in a rearranged region of a G3 individual, two or four signals were expected in the case of a deletion or a translocation (via HE) that had been fixed at the homozygous stage, respectively. In all four cases tested (one EMZ and three RCC plants), four signals were identified in each G3 individual (Figure 6), indicating the presence of fixed translocations (via HE). These rearranged chromosomes harbored a dual color derived from both the C and A chromosomes. For example, the BAC specific to the C02 region deleted in RCC-G3-077 (Figure 6P) hybridized to four regions (red signals), and the Bob014O06 BAC clone specific to the C subgenome only hybridized to half of the C02 chromosome. These results indicated that the 25-Mb fragment lost on C02 was replaced by the homeologous A02 region. Similar observations were clearly observed between the A04 and C04 chromosomes in RCC-G3-055 (Figure 6H), for which a 6-Mb fragment from C04 was replaced with the homeologous A04 region. These BAC-FISH experiments not only facilitated the identification of the molecular mechanisms involved in the loss of certain genomic regions, but also permitted the observation of structural rearrangements that could not be identified using the 60K SNP array (indicated by stars in Figure 6). The rearrangements that were only identified by BAC-FISH may correspond to a translocation (via HE) present at the heterozygous stage.

### The impact of structural rearrangements on gene content

We determined the effect of these large deletions on gene content using the *B. napus* Darmor reference genome composed of 101,040 genes (Chalhoub *et al.* 2014). A mean number of 1912 and 1689 genes were present in the deleted regions of the RCC and EMZ individuals, respectively (for details of the number of genes lost per deleted region, see Table S2), ranging from 0 to 4501 genes in RCC (4.45%: RCC-G3-053), and from 134 to 3595 genes in EMZ (3.56% EMZ-G3-088). As observed for the deletion size, there were more significant deletions in the C subgenome in both RCC (Wilcoxon rank-sum test, *W* = 12,052, *p*-value = 3.343e−05) and EMZ (Wilcoxon rank-sum test, *W* = 9114.5, *p*-value = 0.01721). To assess the real impact of these deletions, the presence of a homeologous gene was determined using the homeologous gene relationship identified by Chalhoub *et al.* (2014). In these homeologous regions, we observed that ∼75% of genes were present on both homeologs, whereas ∼15% of the deleted genes were not present on the homeologous chromosome (for details, see Table S2).

### Putative gene conversion in resynthesized B. napus populations

In almost all G3 genotypes, we observed the presence of a single SNP marker that displayed a loss of one parental allele (mean of three SNPs per plant) on certain chromosomes. Such an allele loss could be derived from a deletion, a small translocation, or a gene conversion. To decipher the type of rearrangement involved, 10 primer pairs surrounding this type of SNP marker were designed. These primers (Table S3) were designed to consistently amplify homeologous regions of identical size (no indels). Direct sequencing of the PCR products obtained for the diploid parents revealed the presence of a polymorphism that could be detected (double peaks) in the resynthesized S0 *B. napus*. In contrast, the G3 allotetraploids presenting a loss of one parental allele did not exhibit any of these double peaks, validating the loss of one parental allele, but preventing the unequivocal determination of the origin of this deleted region (*i.e.*, gene conversion, translocation, or deletion).

## Discussion

To mimic the early generations of a nascent polyploid, and to better understand the major challenges faced by *B. napus* after its formation, two resynthesized allotetraploid *B. napus* populations (“RCC” and “EMZ”) were obtained for the first time by open pollination, enabling outcrossing. We evaluated their fertility, and compared it to that of the population obtained by manual selfing and to natural *B. napus*. Thereafter, in many G3 individuals obtained by open pollination, we evaluated the level of numerical aneuploids, identified SVs at the whole genome level, and determined their impact on the genome size, gene content, allelic diversity, and meiotic stability.

### Aneuploidy and fertility of resynthesized B. napus populations

The level of aneuploid progenies was assessed in our resynthesized *B. napus* plants obtained via open pollination, and we observed a very low percentage of aneuploid progenies (<10%). This level of numerical aneuploids was much smaller than the ∼60% numerical aneuploid plants identified in resynthesized *B. napus* produced by controlled selfing (Xiong *et al.* 2011). In the latter study, there was no selection for plants with 38 chromosomes, similar to our populations obtained by open pollination. The level of aneuploidy observed in our populations obtained by open pollination was similar to those identified in resynthesized AT3 allotetraploid wheat (11.7%) presenting stable karyotypes (Zhang *et al.* 2013a), or in natural populations of *Tragopogon* neoallotetraploids (13.5% in *T. mirus* and 10% in *T. miscellus*) (Chester *et al.* 2013, 2015). Because of the negative impact of numerical aneuploidy on fertility (Xiong *et al.* 2011; Zhang *et al.* 2013b), the contribution of such aneuploids to the production of the following generation after outcrossing will decrease over time, and they will be less prevalent in the population.

The fertility of our two resynthesized *B. napus* populations (obtained after either open pollination or manual selfing) was assessed, and found to be very low compared to natural *B. napus*. It has been established that very frequent homeologous pairing occurs during the first meiosis of a resynthesized *B. napus* S0 plant (Szadkowski *et al.* 2010), generating gametes that carry structural rearrangements. It is likely that some of these latter gametes are not viable, partially explaining the poor seed yield observed in resynthesized *B. napus* compared with natural oilseed rape. After controlled self-fertilization of resynthesized *B. napus*, structural rearrangements with more or fewer deleterious effects will be fixed, decreasing plant fertility. In contrast, open pollination under cages can generate progenies from either the selfing or outcrossing of plants carrying different rearrangements. This latter case will contribute to purging some of the deleterious modifications. Indeed, we observed that the decreased fertility was reduced in plants produced by open pollination compared to controlled selfing. Another factor affecting fertility is related to the origin of the diploid parental progenitors. Indeed, we observed a different level of fertility between our two populations (with EMZ being consistently less fertile than RCC plants). In our study, the *B. oleracea* and *B. rapa* individuals used to create the resynthesized *B. napus* belonged to different subspecies. In addition, the self-incompatibility alleles carried by the parental diploid species will also affect the fertility and the putative successful establishment of a novel polyploid. Indeed, controlled self-fertilization of our two populations was performed, and the EMZ plants were almost sterile, in accordance with the self-incompatibility of EMZ in contrast to the self-compatibility of RCC plants (Hadj-Arab 2011).

To date, it is unknown whether the high fertility of natural *B. napus* resulted from the continuous selection of individuals with the highest karyotype stability and fertility, or to an unknown mechanism leading to the restoration of fertility soon after the formation of *B. napus*.

### Structural rearrangements in resynthesized B. napus populations

Structural rearrangements were evaluated at the whole genome level in G3 resynthesized *B. napus* individuals using the Illumina 60K array. Using this array, a mean number of one SNP marker per 200–300 kb was polymorphic between the diploid parents used to create the resynthesized *B. napus*, enabling a relatively fine identification of SVs. We observed that 32 of the 33 genetically studied G3 plants exhibited important SVs. We were able to determine that the identified fragment losses occurred significantly more on the C than the A-subgenome, which agreed with previous analyses of populations from resynthesized *B. napus* (Song *et al.* 1995; Gaeta *et al.* 2007; Szadkowski *et al.* 2010), or from *B. napus* varieties (Chalhoub *et al.* 2014). For the first time, by analyzing many synthetics derived from two different genetic backgrounds, we were able to determine that these losses may represent up to 5% of the whole genome.

To identify the molecular mechanisms involved in the losses of these genomic regions, BAC-FISH experiments were conducted using four different plants. In all cases, these losses were due to translocations (via HE). Even if all the identified fragment losses were the result of translocations, these structural rearrangements led to the reduced *B. napus* genome size because the lost C genomic regions were replaced by consistently smaller homeologous regions from the A genome. Among our plants obtained via open pollination, we were surprised to identify so many translocated regions that had been fixed at the homozygous stage after only three rounds of meiosis. This result indicates that the translocation of similar genomic regions was a highly frequent event. This observation is consistent with the high frequency of rearranged A01/C01 chromosomes in the gametes (∼50%) of S0 resynthesized *B. napus* plants (Szadkowski *et al.* 2010). In our G3 populations, up to seven rearrangements and losses of up to 35 Mb were detected. However, it is important to note that all the losses observed in our material, and discussed thereafter, were most likely replaced by homeologous regions. Some chromosomes (A01, A09, C01, C02, C03, C04, C06, and CO9) presented genetic changes (losses) in individuals from both populations, while other chromosomes presented fragment losses in only one population (A02, A03, A07, A10, C05, and C08), or never presented any fragment losses in any synthetics. These results are in agreement with those of Gaeta *et al.* (2007), who observed that rearrangements were not randomly distributed across the genome in their aneuploid S5 plants from resynthesized *B. napus*. No fragment losses were observed in the A05, A06, A08, A10, or C07 chromosomes within our G3 populations. Interestingly, whole genome sequencing of five natural *B. napus* varieties also revealed an absence of fragment losses resulting from the homeologous exchange of all these chromosomes (except A05) (Chalhoub *et al.* 2014). The absence of structural variations on these chromosomes may be explained in several ways: (i) these chromosomes exhibit low synteny with their respective homeologous chromosomes (Xiong *et al.* 2011), (ii) there may be hot and cold regions of structural variation, and (iii) the loss of some chromosome regions may be deleterious. In addition to the nonrandom distribution of structural variations between chromosomes, most rearrangements occurred at the distal ends of chromosomes near the telomere or subtelomeric regions where most crossover events occur (Nicolas *et al.* 2012). Only a few rearrangements included the active centromeres of the C01, C02, and C09 chromosomes, which were consistently replaced by the corresponding homeologous centromeres. Overall, our results show that many regions of resynthesized *B. napus* genomes are highly rearranged, and are larger than those described for natural varieties of *B. napus* (Chalhoub *et al.* 2014). This increased genomic stability in natural *B. napus* may be the result of the continuous purging of the most detrimental rearrangements by human and natural selection (Gaeta *et al.* 2007). Another explanation relies on the presence of loci that suppress crossover formation between nonhomologous chromosomes at the time of *B. napus* formation, or soon after, enabling its successful establishment (Jenczewski *et al.* 2013).

The SVs identified in our G3 plants may represent the tip of the iceberg, because only large translocations that were fixed at the homozygous stage, but not those fixed at the heterozygous stage, could be identified using the array (Figure 3). However, these latter rearrangements were observed in few G3 plants using GISH-like experiments (Figure 6). Due to the lower cost and common use of NGS technologies, a wider range of structural variants (indels, inversions, mobile element transpositions, or translocations) will be identified in the near future (Saxena *et al.* 2014; Tattini *et al.* 2015). These technological advances will permit to finely determine how deeply the genome is rearranged, and will allow a better understanding of the molecular mechanisms involved in the evolutionary structural dynamics of nascent allopolyploid *B. napus*.

### The impact of structural rearrangements on genome size, gene content, allelic diversity, and meiotic stability

Among the G3 individuals studied herein, major losses of chromosome regions were observed. For the first time, we could determine the size and gene content of numerous resynthesized plants. We observed that a mean value of ∼4% of the whole genome size of *B. napus* was rearranged in a few generations. Overall, these rearrangements caused gene losses mainly from the C subgenome, with a loss of up to 10% of the genes belonging to the C subgenome. The impact of these deleted genes was most likely buffered by the replacement of most of these genes (77%) by their homeologs present on the A subgenome. Thus, if all the deletions identified in this study were due to translocations (via HE), then the overall gene content will not be dramatically decreased, but will strongly affect the genetic diversity in the rearranged regions. Duplication of a genomic region after HE may either have a detrimental, natural, or beneficial phenotypic effect, as exemplified by the increased seed yield (Osborn *et al.* 2003) or disease resistance (Zhao *et al.* 2006). It has been shown that, for the main agronomical traits, some QTLs are often carried by homeologous regions with different genetic value in *B. napus* (*i.e.*, quantitative blackleg resistance: Fopa Fomeju *et al.* 2014). Thus, the use of resynthesized *B. napus* individuals, which present a highly shuffled genome, will offer a new tactic to test the phenotypic impact of duplicating, or removing, a region carrying a QTL of agronomic interest.

For all genotyped G3 individuals, the meiotic stability was evaluated, which showed that it was strongly affected compared to natural *B. napus*. We compared the meiotic behavior determined in each G3 plant to the observed rearrangements (number, percentage, or position on the chromosome), but did not identify any clear correlation. Further studies are required to establish the relationship among the size, location, and number of translocations with meiotic stability of newly formed *B. napus* populations, and their impact on seed fertility.

In conclusion, our results, mimicking what probably occurred under natural conditions, highlight the extensive shuffling of the *B. napus* genome immediately after allopolyploidization. Overall, the identified structural variations that primarily result from translocations (via HE) led to a decrease in the size, gene content and allelic diversity of the genome. We show that open pollination of the resynthesized *B. napus* individuals within the initial generations enabled the natural selection of individuals with the most stable karyotype, and most likely purged some deleterious rearrangements. However, these resynthesized populations still presented unstable meiotic behavior and low fertility, indicating that further analyses of the structural and functional evolutionary dynamics of resynthesized *B. napus* populations are necessary to improve our current understanding of the key mechanisms involved in meiotic stabilization and the restoration of fertility.

## Supplementary Material

Supplemental material is available online at www.g3journal.org/lookup/suppl/doi:10.1534/g3.116.036517/-/DC1.
