# Supplementary material for: The Impact of Open Pollination on the Structural Evolutionary Dynamics, Meiotic Behavior, and Fertility of Resynthesized Allotetraploid Brassica napus L
Source: G3 (Bethesda). 2016 Dec 21;7(2):705–17. doi: 10.1534/g3.116.036517 (PMC5295613; doi:10.1534/g3.116.036517)
Supplement: Supplementary file 2 [file 705FigureS2.pdf]

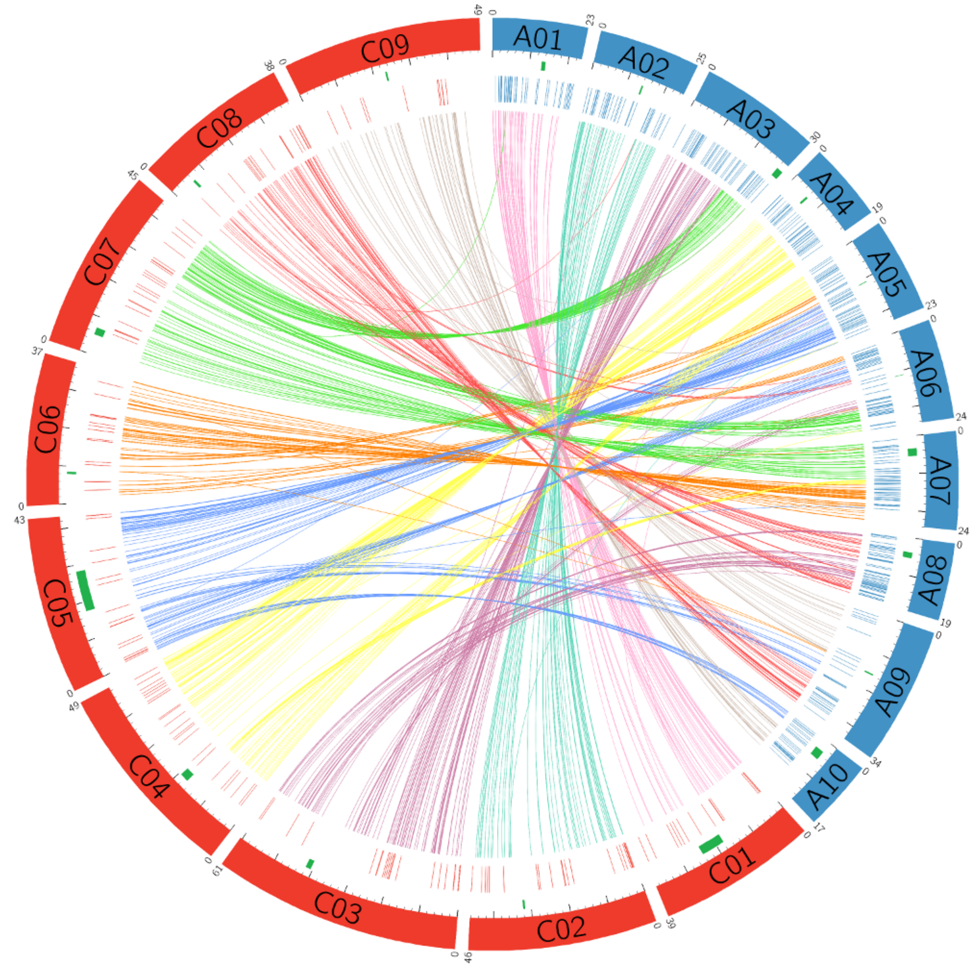

Supplemental Figure S2. Positions on *B. napus* chromosomes (Chalhoub *et al*. 2014) of the polymorphic markers between 'HDEM' and 'Z1'. The *B. napus* chromosomes belonging to the A and C subgenomes are shown in blue and red, respectively. The size of each chromosome (in Mbp) is indicated above each chromosome, and a ruler is drawn underneath each chromosome with larger and smaller tick marks every 10 and 2 Mbp, respectively. The locations of active centromeres (Mason *et al*. 2016) are indicated by green rectangles under each chromosome. The bars indicate the polymorphic markers between 'HDEM' and 'Z1' for which a single hit on the A or C subgenome was obtained using *B. napus* Darmor genome sequence (Chalhoub *et al*. 2014). The links indicate the polymorphic markers between 'HDEM' and 'Z1' for which a single hit on the A and C *B. napus* subgenome was obtained.
