# Supplementary material for: The Impact of Open Pollination on the Structural Evolutionary Dynamics, Meiotic Behavior, and Fertility of Resynthesized Allotetraploid Brassica napus L
Source: G3 (Bethesda). 2016 Dec 21;7(2):705–17. doi: 10.1534/g3.116.036517 (PMC5295613; doi:10.1534/g3.116.036517)
Supplement: Supplementary file 4 [file 705FigureS4.pdf]

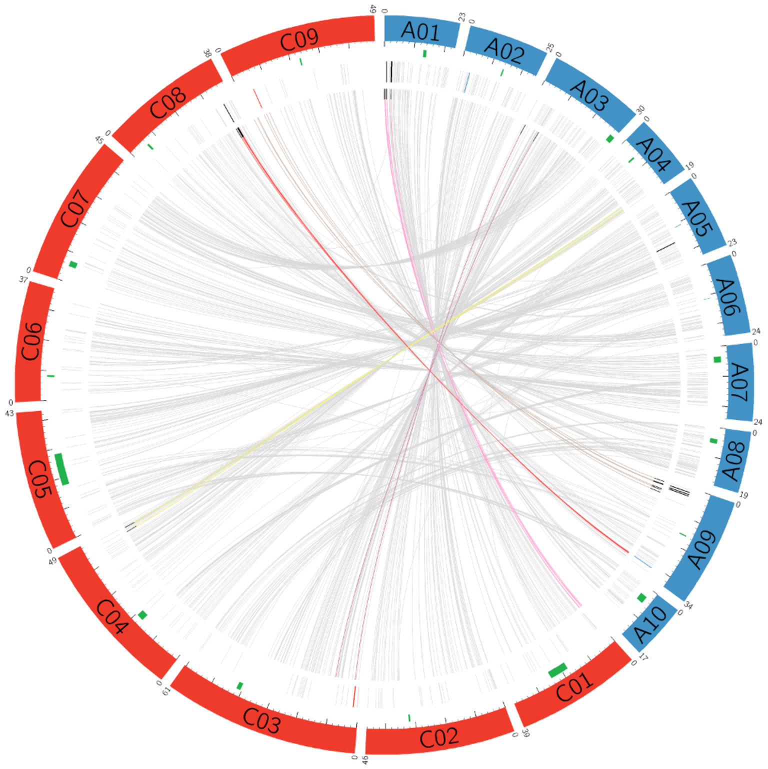

Supplemental Figure S4. Deletions identified in EMZ ind. 078. The deletions identified in EMZ ind. 078 have been represented graphically by Circos (Krzywinski *et al.* 2009). The *B. napus* chromosomes belonging to the A and C subgenomes are shown in blue and red, respectively. The size of each chromosome (in Mbp) is indicated above each chromosome, and a ruler is drawn underneath it with larger and smaller tick marks every 10 and 2 Mbp, respectively. The locations of active centromeres (Mason *et al.* 2016) are indicated by green rectangles under each chromosome. The bars in the first inner circle correspond to the polymorphic markers between the two diploid parental lines. The links in the most inner circle indicate the physical localization (homoeologous relationships) of the markers that were polymorphic between the two diploid parental lines. The gray bars and links (between an A and a C chromosome) indicate the markers for which the two parental alleles were still present in the synthetic plant considered. Conversely, markers presenting a loss of one parental allele are color-coded. A black bar or the black portion at the end of a colored link indicates from which chromosome the allele was lost.
