# Supplementary material for: The Impact of Open Pollination on the Structural Evolutionary Dynamics, Meiotic Behavior, and Fertility of Resynthesized Allotetraploid Brassica napus L
Source: G3 (Bethesda). 2016 Dec 21;7(2):705–17. doi: 10.1534/g3.116.036517 (PMC5295613; doi:10.1534/g3.116.036517)
Supplement: Supplementary file 5 [file 705TableS1.docx]

Table S1. Positions on *B. napus* chromosomes (Chalhoub *et al*. 2014) of the bacterial artificial chromosomes (BACs) used in the BAC-FISH experiment. (.xlsx, 10 KB)

<http://www.g3journal.org/lookup/suppl/doi:10.1534/g3.116.036517/-/DC1/TableS1.xlsx>
