# Supplementary material for: The Impact of Open Pollination on the Structural Evolutionary Dynamics, Meiotic Behavior, and Fertility of Resynthesized Allotetraploid Brassica napus L
Source: G3 (Bethesda). 2016 Dec 21;7(2):705–17. doi: 10.1534/g3.116.036517 (PMC5295613; doi:10.1534/g3.116.036517)
Supplement: Supplementary file 7 [file 705TableS2.docx]

Table S2. Positions on *B. napus* chromosomes of the different deletions observed in each synthetic *B. napus* individual investigated in this study. (.xlsx, 24 KB)

[http://www.g3journal.org/lookup/suppl/doi:10.1534/g3.116.036517/-/DC1/TableS2.xlsx](http://www.g3journal.org/lookup/suppl/doi:10.1534/g3.116.036517/-/DC1/TableS1.xlsx)
