# Supplementary material for: The Impact of Open Pollination on the Structural Evolutionary Dynamics, Meiotic Behavior, and Fertility of Resynthesized Allotetraploid Brassica napus L
Source: G3 (Bethesda). 2016 Dec 21;7(2):705–17. doi: 10.1534/g3.116.036517 (PMC5295613; doi:10.1534/g3.116.036517)
Supplement: Supplementary file 9 [file 705TableS3.docx]

Table S3. List of the primer pairs designed to determine the presence of putative genic conversion in some synthetic *B. napus* individuals. (.xlsx, 14 KB)

<http://www.g3journal.org/lookup/suppl/doi:10.1534/g3.116.036517/-/DC1/TableS3.xlsx>
